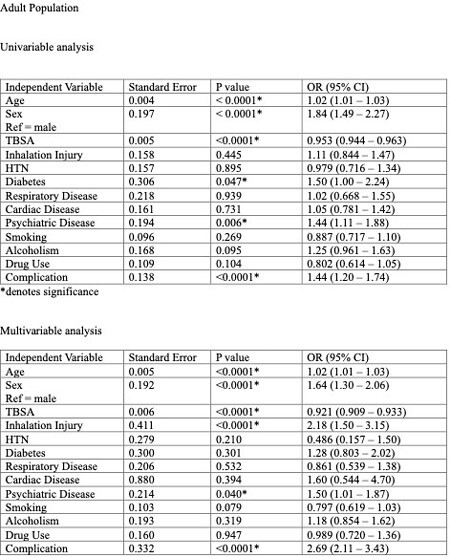# 126 Predictors of Lengthened Admission in Adult Burn Patients, a Secondary Analysis of 1796 Cases

**DOI:** 10.1093/jbcr/irae036.125

**Published:** 2024-04-17

**Authors:** Xi Ming Zhu, Diana Tedesco, Lucas Gallo, Shahriar Shahrokhi, Marc G Jeschke

**Affiliations:** McMaster University, Hamilton, ON; Hamilton Health Sciences Corporation, Hamilton, ON; Hamilton Health Sciences, Hamilton, ON; McMaster University, Hamilton, ON; Hamilton Health Sciences Corporation, Hamilton, ON; Hamilton Health Sciences, Hamilton, ON; McMaster University, Hamilton, ON; Hamilton Health Sciences Corporation, Hamilton, ON; Hamilton Health Sciences, Hamilton, ON; McMaster University, Hamilton, ON; Hamilton Health Sciences Corporation, Hamilton, ON; Hamilton Health Sciences, Hamilton, ON; McMaster University, Hamilton, ON; Hamilton Health Sciences Corporation, Hamilton, ON; Hamilton Health Sciences, Hamilton, ON

## Abstract

**Introduction:**

Existing research has examined the relationship between the amount of total body surface area (TBSA) burn and length of stay (LOS). As a result, the conventional ratio of 1day LOS/1% TBSA ratio has been updated to 1.5days LOS/1% TBSA. In this study, we aim to elucidate patient and injury characteristics that affect our prognostic indicator, leading to a prolonged LOS.

**Methods:**

This was a secondary analysis of a cohort study of surviving patients admitted to a tertiary adult burn center between January 1, 2006, and June 30, 2021. Adult patients less than 60 years of age were stratified into expected LOS ( < 1.5 days/%TBSA) and greater than expected LOS (>1.5 days days/%TBSA). Patient demographics, TBSA, burn etiology, inhalation injury, pre-admission co-morbidities, and in-hospital complications were tabulated Logistic regression was performed using IBM SPSS Statistics 29 and Stata Statistical Software: Release 18.

**Results:**

1796 patients with a mean age of 39 years were included for analysis of the adult population. 905 patients had the expected LOS/TBSA ratio, and 891 exceeded this ratio. Univariable analysis indicated patients with greater age [1.02 (1.01 – 1.03) p < 0.0001], female sex [1.84 (1.49 – 2.27) p < 0.001], diabetes [1.50 (1.00 – 2.24) p = 0.047], psychiatric illness [1.44 (1.11 – 1.88) p = 0.006], and those who experienced complications (such as infection, graft failure, pneumonia, sepsis) [1.44 (1.20 – 1.74) p < 0.0001] during admission were more likely to exceed their predicted LOS.

Multivariate analysis found that greater age [1.02 (1.01 – 1.03) p < 0.0001], female sex [1.64 (1.30 – 2.06) p < 0.0001], inhalation injury [2.18 (1.50 – 3.15) p < 0.0001], psychiatric illness [1.50 (1.01 – 1.87) p = 0.04], and complications [2.69 (2.11 – 3.43) p < 0.0001] were contributing factors that increased the likelihood of patients exceeding predicted LOS. Diabetes was not a statistically significant contributor.

**Conclusions:**

Progress has been made to identify patient and injury factors that increase the likelihood of increased LOS for adult patients with burn injuries. This provides valuable data for physicians to better assess patients and improve their quality of care.

**Applicability of Research to Practice:**

Identification of key burn patient characteristics aids in prognostication. Determining length of stay aids with coordination of patient care with the multi-disciplinary team.